# Exploring the Properties of Curved Lipid Membranes: Comparative Analysis of Atomistic and Coarse-Grained Force Fields

**DOI:** 10.1021/acs.jpcb.4c02310

**Published:** 2024-07-11

**Authors:** Maria Domanska, Piotr Setny

**Affiliations:** Centre of New Technologies, University of Warsaw, Banacha 2c, 02-097 Warsaw, Poland

## Abstract

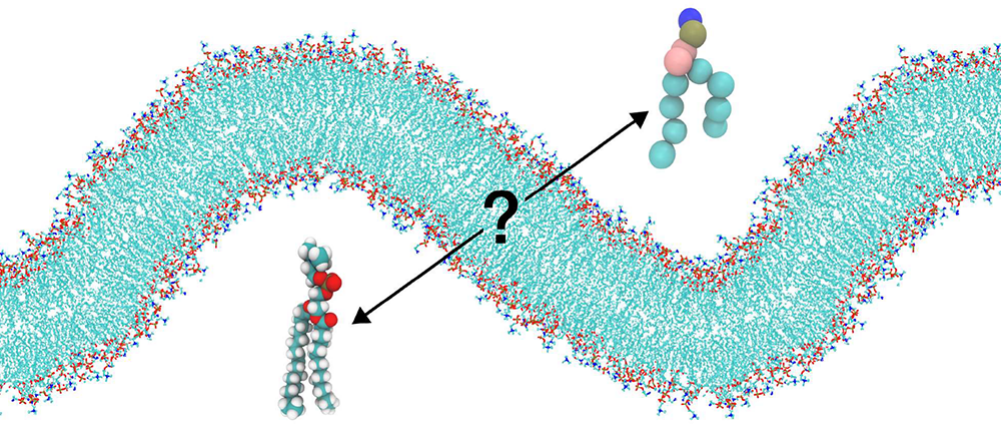

Curvature emerges as a fundamental membrane characteristic crucial for diverse biological processes, including vesicle formation, cell signaling, and membrane trafficking. Increasingly valuable insights into atomistic details governing curvature-dependent membrane properties are provided by computer simulations. Nevertheless, the underlying force field models are conventionally calibrated and tested in relation to experimentally derived parameters of planar bilayers, thereby leaving uncertainties concerning their consistency in reproducing curved lipid systems. In this study we compare the depiction of buckled phosphatidylcholine (POPC) and POPC–cholesterol membranes by four popular force field models. Aside from agreement with respect to general trends in curvature dependence of a number of parameters, we observe a few qualitative differences. Among the most prominent ones is the difference between atomistic and coarse grained force fields in their representation of relative compressibility of the polar headgroup region and hydrophobic lipid core. Through a number of downstream effects, this discrepancy can influence the way in which curvature modulates the behavior of membrane bound proteins depending on the adopted simulation model.

## 1 Introduction

Biological membranes are crucial for the functioning of the cell. They serve as physical barriers between cellular compartments,^1^ and changes in their dynamics and topography play an important role in a number of processes, such as cellular trafficking,^2^ signal transduction,^3^ fusion,^4^ fission^5^ and organelle shaping. Living cells contain regions of lipid bilayers presenting persistent curvature of varying degrees.^6^ Namely, extracellular vesicles and the Golgi apparatus tubules can have curvature radii as small as 15 nm.^6,7^ In turn, the curvature radius of the inner mitochondrial membrane is 20 nm, whereas in the case of filopodia it reaches 100 nm.^6,8^ This wide spectrum of curvatures is generated as a result of the interplay between the elastic properties of the lipid bilayer, the intrinsic asymmetry of lipids, and membrane modeling by specialized proteins.^9^ Furthermore, a number of proteins aid in the organization of higher level structures involving distinct membrane topography by adopting specific locations owing to their curvature-sensing capabilities.^10−13^

Molecular dynamics (MD) simulations are a valuable tool for investigating mechanistic driving forces that govern the complex behavior of membrane systems. To realistically simulate biological membranes, however, the significant complexity and heterogenity of its building blocks has to be taken into account, as well as their properly balanced mutual interactions. Accordingly, the ability to capture the nuanced nature of lipid membranes critically depends on the choice of simulation parameters.^14^ To this end, a number of models have been developed that offer varying levels of structural resolution and accessible time scales. All atom (AA) force fields for lipids, such as CHARMM (Charmm36m^15^), Amber (Lipid 21^16^), SLipids,^17^ and OPLS-AA,^18^ provide a high degree of structural detail, however, at a considerable computational cost that constraints the feasible system sizes and limits the duration of simulations. United atom force fields, including GROMOS,^19^ OPLS-UA,^20^ TraPPE-UA,^21^ and C36-UA,^22^ offer higher efficiency by collapsing nonpolar hydrogen atoms onto the heavy atoms to which they are connected. Finally, the most computationally efficient but least detailed molecular representation is provided by coarse-grained (CG) models in which individual interaction sites, so-called beads, are used to represent several (3–6) heavy atoms. CG force fields are exemplified by MARTINI,^23^ SPICA (formerly Shinoda/Devane/Klein (SDK)),^24^ the SIRAH,^25^ and ELBA force fields.^14,26^

To date, a number of studies evaluating the performance of available models in reproducing lipid membrane properties have been conducted.^14,27−34^ In general, the most popular families of current force fields, such as Amber, CHARMM, and Martini, are observed to capture the structural features of flat membrane patches in good agreement with experimentally derived parameters. Also satisfactory, in particular in the case of atomistic models, is the representation of more challenging properties, such as elastic constants, lipid self-diffusion, or temperature-dependent phase transitions. There are still, however, membrane characteristics, mainly related to the description of the hydrophilic lipid moieties, whose agreement with experiments is less consistent, namely interactions of lipid headgroups with cholesterol^35,36^ and proteins^37−39^ or the binding of cations to PC headgroups.^40^ Moreover, it has been noted that even small changes in parameters of MD simulations (e.g., cutoff distance applied to dispersion interactions) can lead to pronounced changes in the state of the examined membrane systems.^27^

Constant advances in computational power, allowing simulations of increasingly complex systems, have been naturally adopted for the studies of nonflat membrane topography,^9^ with particular focus on uncovering the details of curvature generation and sensing by proteins.^41−43^ Still, the construction of systems comprising stable and controllable curved membrane patches is not straightforward and requires specialized techniques. One kind of approach pursued in this respect is based on mimicking naturally curved systems. They can rely on stimulated clustering of lipids with particular packing preferences at one bilayer leaflet^34,44^ or the presence of membrane-modeling proteins.^45−47^ In addition, particular membrane constructs can be considered such as easily deformable bicelles^48−51^ or intrinsically curved vesicles^52^ that can be assembled using a set of dedicated modeling tools.^53−57^ Another kind of approach involves additional potentials that impose membrane bending. Possibilities here range from virtual beads that are fixed in space and repel or attract lipid molecules,^58^ to artificial, perforated solid support,^34,59,60^ to collective variables encoding the desired shape.^6,34^ A popular and efficient approach is based on a simulated buckling technique.^61−63^ A buckled membrane is obtained when an elongated fragment of a flat bilayer with a fixed width (e.g., in the *y*-direction) is gradually compressed along its longer dimension (e.g., *x*) such that it generates a sinusoidal standing wave, continuous in periodic boundary conditions. Once the desired geometry is achieved, the simulation box size in the *xy*-plane becomes fixed to ensure that the membrane will maintain the curved geometry.

A fast growing number of computational studies involving nonflat membrane topography calls for critical assessment of popular simulation models’ abilities to consistently represent lipid behavior under curvature induced stress. Given the relatively limited experimental characterization of the microscopic properties of bent membranes, it is of interest to investigate the agreement between possibly independent atomistic force fields and to verify if such established baseline is reproduced at the coarse grained level, which is typically utilized in the case of large systems with complex bilayer geometries.

To this end, we focus on four popular models: atomistic, which includes the Amber Lipid21^16^ and Charmm36^15^ force fields, and coarse grained, which are represented by the Martini 2 and 3 force fields.^23,64^ We aim to investigate the curvature dependence of selected membrane properties that are often considered in the context of force field validation for flat geometries. We primarily consider one component, the phosphocholine membrane, as this lipid type is among the most widespread in computer simulations. In addition, we investigate a two component model which includes cholesterol, a ubiquitous lipid whose preference to negative curvature is at the heart of a number of biological effects.

## 2 Methods

### 2.1 System Description

We considered two kinds of membrane systems: (a) a one component bilayer, composed of 1-palmitoyl-2-oleoyl-*sn*-glycero-3-phosphocholine (POPC) molecules, and (b) a two component bilayer composed of POPC and cholesterol (CHL) molecules at 0.6:0.4 mole fractions. The systems were parametrized using Amber Lipid21 (L21),^16^ Charmm36m (C36),^15^ Martini 2 (M2),^64^ and Martini 3 (M3)^23^ force fields. For the latter, a recently published parameter set for CHL was adopted.^65^ In addition to curved membranes, we simulated flat POPC membrane patches to verify system setup and analysis methods. A detailed summary of the systems under study is provided in Tables S1 and S2.

### 2.2 System Preparation

All systems were initially constructed using the CHARMM-GUI Membrane Builder Tool^66^ as square bilayers hydrated with 2.25 and 3.5 nm of 0.15 M Na^+^, Cl^–^ aqueous solution layers for flat and buckled systems, respectively. Detailed composition of each system is provided in Table S1. Preliminary equilibration runs were carried out according to the default CHARMM-GUI protocol. Then, in order to generate elongated membranes, respective systems were first compressed along the *y*-axis by increasing the corresponding component of the pressure tensor (to 5 bar (CG) or 10 bar (AA)) and carrying out simulations until the length of the simulation box in this direction, *L*_*y*_, contracted to  nm. Subsequently, the *y*-dimension of the simulation box was fixed, and the systems were compressed along the *x*-axis from  nm to reach a *L*_*x*_ ≈ 24 nm box length in this direction. The resulting compression factor for the buckled bilayer, defined as , was in the order of 0.2. Following the above procedure, the *x*-dimension of the simulation box was also fixed and buckled systems were first equilibrated at *T* = 350 K for 3.5 μs, cooled down to *T* = 310 K, and equilibrated further for 1.6 and 6 μs for atomistic and coarse grained systems, respectively. Final production runs extended over 1 μs (AA systems) and 5 μs (CG systems). All simulations were carried out using the GROMACS 2018.7 and 2022.3 software.^67^ The molecular dynamics setups used for final equilibration and production runs are outlined in Table 1.

**Table 1:** Parameters Used in MD Simulations^a^

parameter	L21	C36	M2	M3
time step (fs)	2	2	20	20
*R*_Coulomb_ (nm)	1.0	1.2	1.1	1.1
Coulomb method	PME	PME	RF	RF
*R*_vdW_ (nm)	1.0	1.0–1.2	1.1	1.1
vdW method	VCO + PSV + DC	VCO + FS	VCO + PSV	VCO + PSV
temperature (K)	310	310	310	310
pressure (atm)	1	1	1	1
barostat	PR	PR	PR	PR
thermostat	NH	NH	VR	VR
water model	TIP3P	TIP3P	CG	CG
constraints	H-bonds	H-bonds	–	–

a Abbreviations used: PR, Parrinello–Rahman; NH, Nosé–Hoover; PME, particle mesh Ewald; TIP3P, transferable intermolecular potential with three points;^68^ vdW, van der Waals; VR, velocity rescale; RF, reaction-field; CG, coarse grained water model; VCO, Verlet cutoff; DC, dispersion correction (EnerPress); FS, force-switch; PSV, potential-shift-Verlet.

### 2.3 Analysis

All analysis of buckled membrane systems was based on the last 1 or 5 μs of production runs for AA and CG simulations, respectively. Uncertainties were estimated as the standard error of the mean using block averaging after splitting the analyzed trajectory parts into four even blocks. Calculations were carried out with custom Python scripts with the use of the MDAnalysis library^69^ and GROMACS analysis tools. All reference values obtained for *K* = 0 nm^–1^ are listed in Table 2.

**Table 2:** Numerical Values for *K* = 0 nm^–1^ Obtained from Buckled Membrane Simulations^a^

parameter	L21	C36	M2	M3	experiment
POPC					
*D*_PP_ (nm)	3.86	3.90	3.88	3.83	3.87^b^
APL (nm^2^)	0.64	0.64	0.65	0.65	0.65^b^,^72^ 0.66^c^
*S*_CC_ S_N_1	0.38	0.40	0.28	0.28	
*S*_CC_ S_N_2	0.34	0.38	0.34	0.37	
*S*_CH_ S_N_1	0.15	0.17	–	–	
*S*_CH_ S_N_2	0.11	0.13	–	–	
*H*_H_ (*n*H_2_O/L)	6.95	6.98	–	–	
*H*_T_ (*n*H_2_O/L)	0.0003	0.0002	–	–	
*z*_p_ (nm)	1.20	1.37	0.99	1.02	
2*z*_p_/*D*_PP_	0.62	0.71	0.51	0.53	0.67^74,75^
*D* (10^–7^ cm^2^/s)	0.62	0.85	4.81	4.71	0.51^b^,^76^1.26^d^

POPC–CHL					
ϕ_CHL_	0.40	0.40	0.40	0.41	
*D*_PP_ (nm)	4.41	4.58	4.12	4.11	4.25^e^
*K*_CHL_^0^ (nm^–1^)	–0.36	–0.38	–0.14	–0.17	–0.34,^78^ −0.49^79^

a Uncertainties and respective values from flat membrane simulations are provided in Tables S5 and S6 and Figure S2.

b interpolated for *T* = 310 K based on data for *T* = 303 K and *T* = 323 K.

c *T* = 310 K.

d *T* = 308 K.

e *T* = 300 K.

#### 2.3.1 Membrane Shape Approximation

In order to assess the local membrane curvature at each simulation frame, we approximated a buckled shape along the *x*-direction with linear combination of six sinus functions:
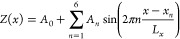
1where {*A*_0_, ..., *A*_*n*_, *x*_*n*_} are parameters.

Their values were obtained by fitting the above equation to a set of (*x*_*i*_, *z*_*i*_) positions occupied by specific lipid atoms or beads. Terminal, non-hydrogen interaction centers of POPC acyl chains were used to estimate the bilayer midplane while phosphorus atoms were used to estimate monolayer surfaces. The number of six sinus components was chosen based on the analysis of how well the obtained curves represent the underlying distributions of atomic positions (see Figure S1 for details).

**Figure 1:**
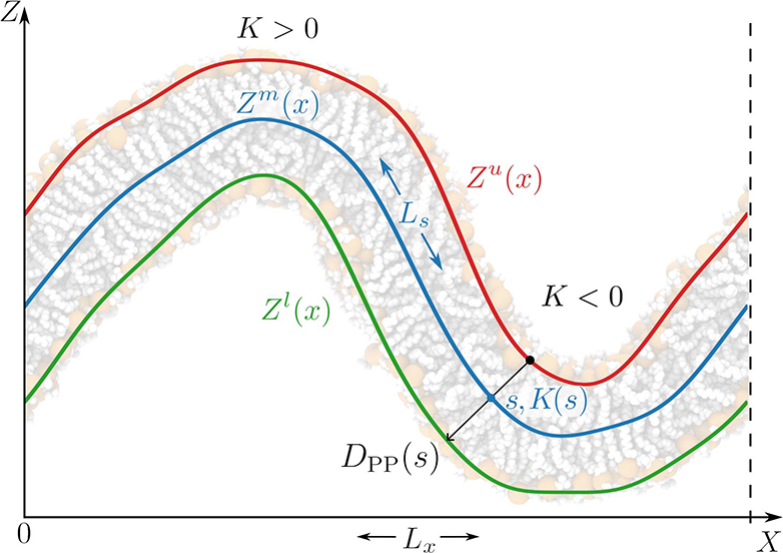
Scheme of curves fitted to the bilayer model for analysis. *Z*^u^, *Z*^l^, and *Z*^m^ are the “upper” and “lower” membrane–water interface and membrane midplane, respectively. *D*_PP_(*s*) is local membrane thickness, assigned to curvature *K*(*s*).

Local membrane curvature at a desired point, *K*(*x*), was evaluated based on curve *Z*(*x*) corresponding to the bilayer midplane and its two first derivatives as
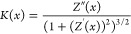
2For the calculation of local membrane properties, curves *Z*(*x*) were discretized into points, {*s*}, spaced by constant arc lengths of δ*s* = 0.1 nm. Since each point *s*_*i*_ has unique coordinates, (*x*_*i*_, *Z*(*x*_*i*_)), in the following we use implicit notation *K*(*s*_*i*_) ≡ *K*(*x*_*i*_) (Figure 1).

#### 2.3.2 Membrane Thickness

Given three sets of points {*s*^u^}, {*s*^l^}, and {*s*^m^} evenly spaced along curves *Z*^u^(*x*), *Z*^l^(*x*), and *Z*^m^(*x*) fitted to phosphorus atoms of the “upper” (u) and “lower” (l) leaflets and membrane midplane (m), respectively, for each point  and  we determined the closest point from the opposing leaflet. The length of the corresponding segment was then assigned as a local membrane thickness, *D*_PP_, to a curvature evaluated at point  that was found to be the shortest distance from this segment (Figure 1).

#### 2.3.3 Bilayer and Monolayer Area Densities

To assess bilayer and monolayer number area densities as a function of curvature, ρ(*K*), for each simulation frame we binned the positions of terminal non-hydrogen atoms or beads of POPC acyl chains according to their closest distances in the *xz*-plane to particular points . Local area densities were then obtained based on the normalized (by the number of centers per lipid) numbers of counts, *N*, per area δ*S* = *L*_*y*_δ*s*, where *L*_*y*_ is the simulation box size along the *y*-axis: . Contributions to bilayer density were averaged according to counts from both membrane leaflets and absolute curvature  assigned to each area, whereas contributions from single leaflets together with respective signed curvatures, , were used for monolayers, assuming the convention that *K* > 0 for monolayer regions with convex lipid–water interfaces.

#### 2.3.4 Interface Area Per Lipid

The interface area per lipid was evaluated by binning the positions of the phosphorus atoms in the *xz*-plane to their closest  or  points from respective monolayers and assuming the area of each bin is equal to δ*S*. The curvature associated with each bin at a given simulation frame was determined as the midplane curvature at the point , closest to the respective bin center.

#### 2.3.5 Lipid Hydration

Curvature-dependent lipid hydration was analyzed separately for the interfacial area and for the hydrophobic membrane core. The number of water oxygen atoms within 0.3 nm from heavy atoms constituting the head or terminal tail region of the POPC molecule, *H*_H_ and *H*_T_, respectively, was calculated for each lipid and was subjected to averaging over the simulation time in the curvature-dependent manner. An instantaneous curvature corresponding to each lipid was established as the midplane curvature at the point *s*^m^, that was closest to the respective phosphorus atom. The cutoff of 0.3 nm was introduced based on the position of the first minimum in the radial distribution function of water oxygen atoms around lipid heavy atoms. A detailed list of lipid atoms used to define head and tail regions is provided in Table S4.

#### 2.3.6 Monolayer Pivotal Plane

To estimate the position of the monolayer pivotal plane, that is the surface within the monolayer core, at which lipid lateral density does not change upon bending, we first assessed curvature dependencies of number area densities, ρ_*A*_(*K*), for each heavy atom, *A* (AA representations) or bead (CG representations) of POPC molecules along curved surfaces fitted to their respective positions in the *xz*-plane. In principle, for atoms located closer to the lipid–water interface than the pivotal plane, ρ_*A*_(*K*) is expected to decrease along with increasing *K*, whereas the opposite trend is expected for atoms located deeper than the pivotal plane (Figure S2). To quantify this dependence, we considered slopes *a*_*A*_ of linear functions fitted to ρ_*A*_(*K*) for each atom over *K* ranging from −0.15 to 0.15 nm^–1^. Finally, the membrane depth *z*_*p*_ corresponding to the pivotal plane was determined as the zero point of the linear function fitted to the set of (*z*_*A*_, *a*_*A*_) points, with *z*_*A*_ being the average depth of the considered POPC atoms or beads obtained from simulations of a flat bilayer (Figure S3).

#### 2.3.7 Lipid Order Parameters

The ordering of lipid hydrocarbon chains was evaluated based on the angle, θ_CX_, of specific vectors: carbon–carbon in the case of *S*_CC_ and carbon–hydrogen in the case of *S*_CH_ order parameters, in relation to the local bilayer normal, **n**(*s*), as
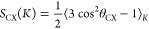
3Here, ⟨·⟩_*K*_ denotes an average over simulation frames for lipids found at membrane locations with curvature *K*(*s*). Having mapped each lipid into a particular point  at the bilayer midplane according to the proximity of its phosphorus atom in the *xz*-plane, local curvature and normal vector orientation were evaluated as  and , respectively. For practical purposes, instead of considering individual  for every POPC molecule, lipids were grouped according to local curvatures at the given simulation frame, and their coordinates were rotated along the *y*-axis by an angle α_*i*_ equal to that between  and the *z*-axis. Subsequently, order parameters were evaluated using standard software^70^ and the LiPyphilic library^71^ for *S*_CH_ and *S*_CC_, respectively. A detailed definition of atomistic carbon–carbon vectors for the calculation of *S*_CC_ parameters consistent with coarse grained representation is provided in Table S3.

#### 2.3.8 Lipid Lateral Diffusion

The curvature-dependent lateral diffusion coefficient *D*(*K*) was estimated by tracing one-dimensional movement of phosphorus atoms along the curved interface surfaces. At each simulation frame, every lipid molecule was labeled according to the local curvature at the position of its phosphorus atom, *K*(*s*_0_) determined as above, and the displacement δ*s* = *s*(*t*) – *s*_0_ was monitored for a subsequent 10 ns. The diffusion coefficient was estimated using the following linear relation:
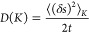
4with an average ⟨.⟩_*K*_ taken over lipids labeled with particular *K* and a fit carried out for *t* between 5 and 10 ns.

#### 2.3.9 Cholesterol Enhancement Ratio

In order to quantify the strength of coupling between cholesterol concentration and local monolayer curvature, we followed the approach proposed by Elías-Wolff at al.^63^ Briefly, given a lipid monolayer formed as a binary mixture of two components, *a* and *b*, with molar fractions ϕ_*a*_ and ϕ_*b*_ and spontaneous curvatures  and , respectively, one can formulate a Helfrich-type free energy functional:
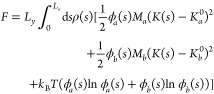
5where *L*_*s*_ is the length of the curved bilayer, *M*_*a*_ and *M*_*b*_ denote bending moduli per lipid molecule of a given type, and *k*_B_*T* is the Boltzmann constant times temperature. The first two terms express the free energy penalty upon departure from each lipid spontaneous curvature, and the last term is a simplified, ideal gas-like mixing entropy. Minimization of the above functional can be conducted subject to constraints governing local composition, ϕ_*a*_(*s*) + ϕ_*b*_(*s*) = 1, and the total number of compounds, , providing equilibrium, position-dependent molar fractions of each component. Acknowledging that at any location along the bilayer midplane, curvatures in the opposite leaflets have the same absolute value but opposite signs, one can obtain a linear relation between so-called enhancement ratio, that is the logarithm of ratios of curvature-dependent local lipid concentrations in the upper and the lower bilayer leaflets (ϕ^u^ and ϕ^l^), and the curvature itself:
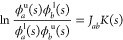
6where , with .

## 3 Results

The curvature dependence of membrane properties was evaluated and compared across four considered force fields. Where possible, the results were validated against experimental findings, including values available for flat membranes.

### 3.1 Membrane Thickness

**Figure 2:**
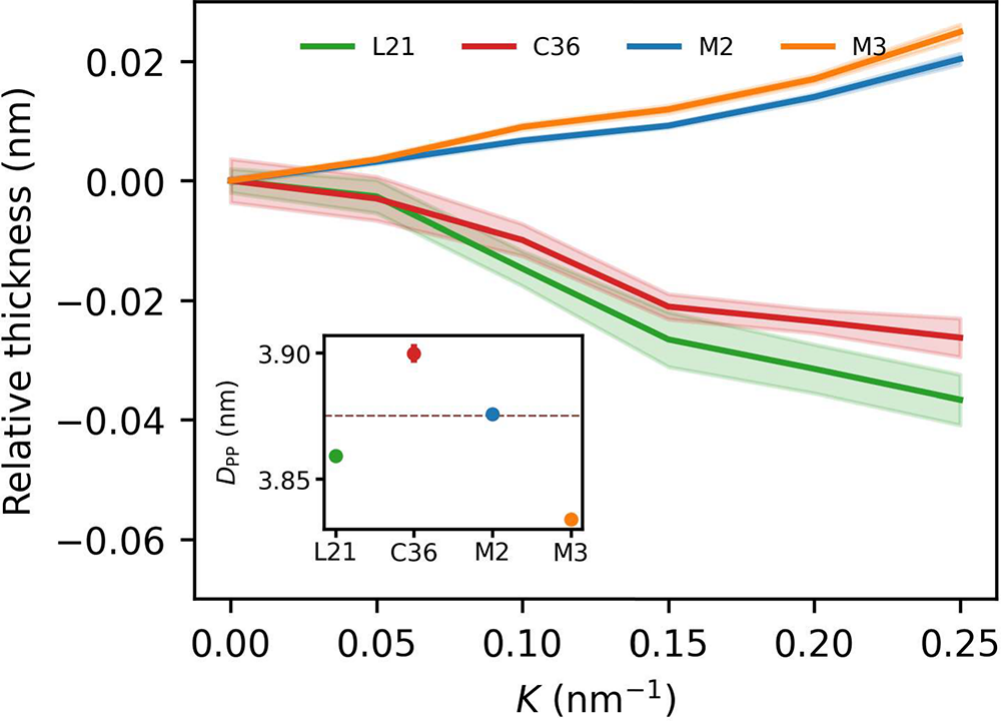
Changes in POPC membrane thickness in relation to curvature. All plots were shifted by reference values at *K* = 0 nm^–1^ (inset). Dashed line in the inset denotes an experimental value for the POPC bilayer (Table 2). Lines are a guide to the eye, and shaded areas correspond to calculated uncertainties.

All considered force fields are known to reproduce the experimental value for flat POPC membrane thickness with less than 0.1 nm deviation.^16,28,30,80^ We obtained similar results in preliminary simulations of flat membrane patches that were used to validate MD setups (Table S6). In order to handle curved bilayers, in the following we consider thickness, *D*_PP_, measured between smooth surfaces, and *Z*^u^(*x*) and *Z*^l^(*x*), fitted to phosphorus atom positions at the *xz*-plane in each of the two membrane leaflets (see the Methods for details). The reference thickness at zero curvature, evaluated in this manner, agrees well with the one that was measured between peaks of phosphorus atom densities in entirely flat membranes (Table S6). Upon leaving the flat regime, membrane thickness appears to generally remain within 0.05 nm from the reference values at *K* = 0 nm^–1^ (Figure 2). Nonetheless, as the curvature increases, clear monotonic trends in membrane thickness can be observed. In this respect, AA force fields reveal a tendency to produce a somewhat thinner POPC bilayer in curved areas, which is qualitatively different to the behavior of CG force fields. Assuming constant membrane volume density, this effect might be consistent with a slight preference of atomistically represented POPC lipids to localize within flat membrane regions, in agreement with their small estimated spontaneous curvature (−0.02 to −0.03 nm^–1^).^79,80^

### 3.2 Bilayer Area Density

**Figure 3:**
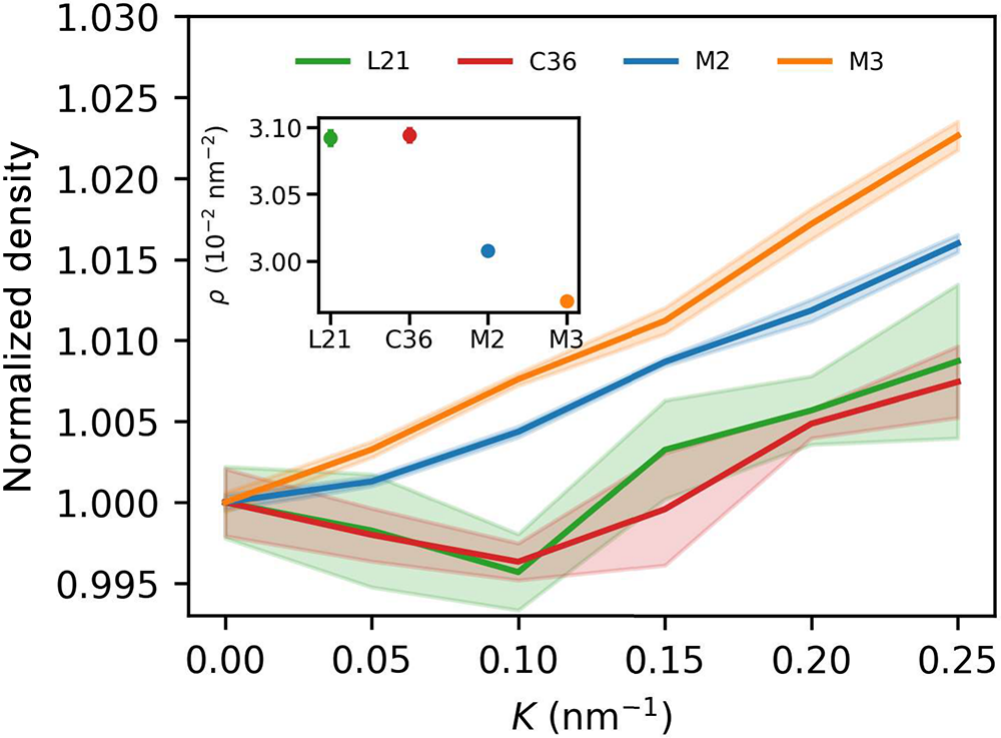
Curvature-dependent POPC bilayer area density. All values were normalized to 1 at *K* = 0 nm^–1^. Inset: absolute area densities.

Curvature-dependent bilayer area density (Figure 3) reflects the distribution of the innermost lipid heavy atoms (or beads) contributed to by both membrane leaflets along the bilayer midplane. Based on our simulations, it appears to vary rather little with respect to flat membrane area density in the case of AA force fields (less than 0.5%) and somewhat more for CG models (up to 2% for the largest considered curvature). Notably, curvature-dependent density changes are again qualitatively different between the two kinds of system representation. AA resolved membranes consistently show a local density maximum at *K* = 0 nm^–1^ and a slight depletion around *K* ≈ 0.1 nm^–1^, whereas CG systems have a definite global minimum at the flat region. These observations support the notion that AA but not CG models capture a slight preference of the POPC lipids to almost flat membrane geometries. The origin of area density increase for *K* > 0.1 nm^–1^ revealed in AA simulations is not clear but might result from a qualitative change in lipid packing upon exceeding critical deformation level.

### 3.3 Lipid Order

A more direct insight into lipid packing behavior is provided by the analysis of curvature dependence of the order parameters. In this respect, as opposed to bilayer thickness and density, which were attributed to the membrane as a whole, we consider separate monolayers. Accordingly, we discriminate between positively and negatively curved leaflets, defined as those having a convex and concave lipid–water interface, respectively (Figure 1).

**Figure 4:**
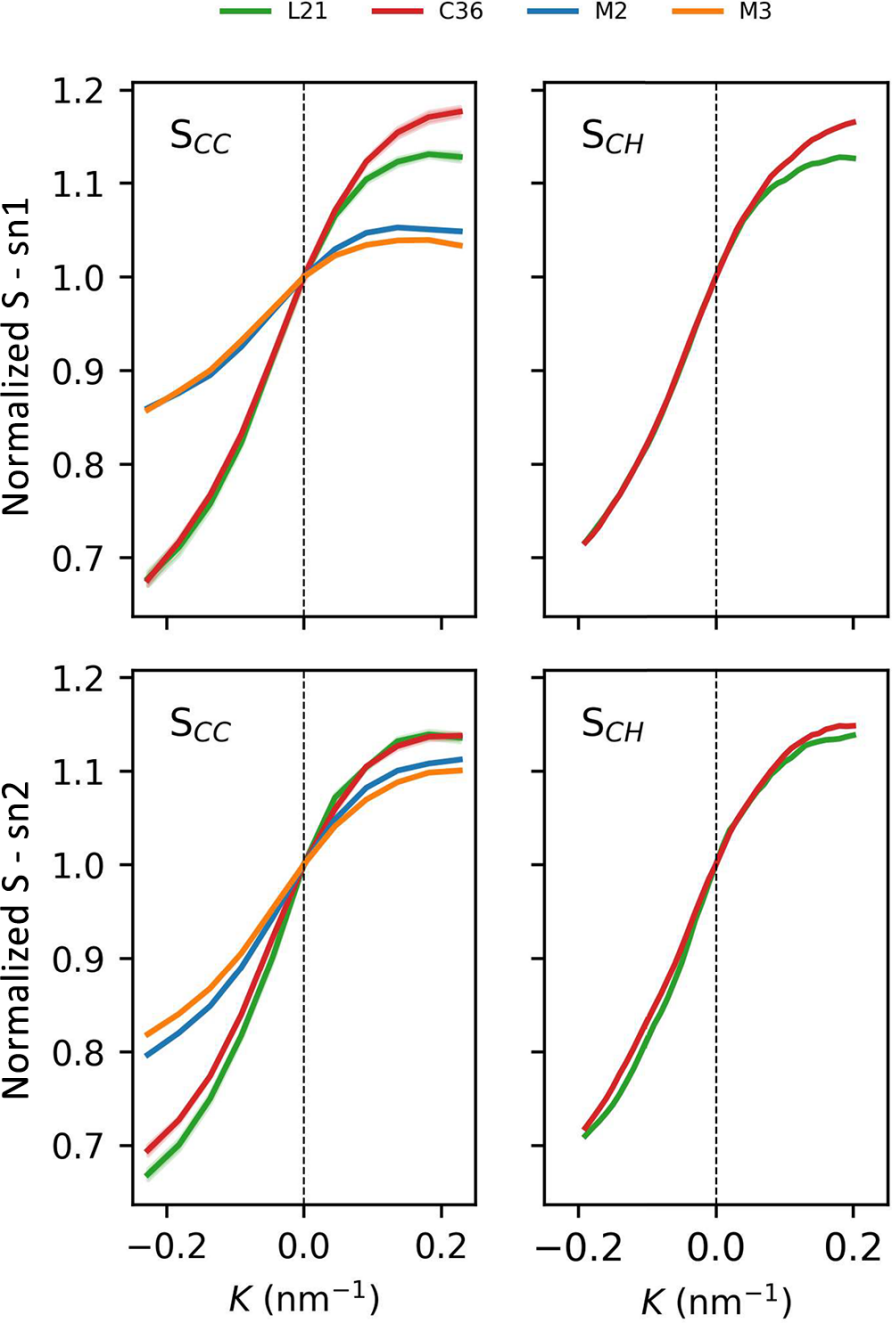
Relative changes of lipid order parameters as a function of membrane curvature. Absolute values at *K* = 0 nm^–1^ are provided in Table 2.

Acyl chain ordering described by an average coarse grained order parameter, *S*_CC_ (see the Methods for details), is clearly increasing with curvature, with all force fields showing qualitatively the same behavior (Figure 4). This result may be explained by the fact that monolayer bending in concave areas leads to crowding of lipid heads but at the same time allows more freedom for acyl chains. On the other hand, increased distance between lipid polar heads in convex areas leads to lateral compression and decreased freedom within the hydrophobic part. Both effects, but in particular the former one, appear to saturate at |*K*| ≈ 0.15 nm^–1^, which possibly indicates a transition to different monolayer bending regimes.

The amplitude of *S*_CC_ changes across the considered curvature range and is evidently larger in the case of AA force fields, with the C36 force field showing a somewhat stronger relative ordering of saturated, S_N_1 acyl chains at convex monolayers compared to the L21 force field. Notably, the absolute *S*_CC_ values achieved at *K* = 0 nm^–1^ (Table 2) do not indicate any greater ordering of CG lipids in comparison to their AA resolved counterparts. It leads to a conclusion that CG parametrization reasonably captures the level of ordering at flat membrane regimes but apparently does not provide enough degrees of freedom to reproduce adequate responses to lateral (de)compression of the membrane core upon bending. Perhaps, this may be a factor contributing to the problems of Martini force fields in sustaining a stable ripple phase reported recently.^81^ Their relatively high degree of lipid tails order that persists under concave interface areas may not allow efficient interdigitation of the two leaflets, which is a necessary prerequisite of ripples formation.

Atomistic *S*_CH_ order parameters, accessible only in the case of L21 and C36 force fields, reveal similar curvature dependence to *S*_CC_ parameters described above (Figure 4). Of note is the fact that a tendency of the C36 model to produce somewhat more ordered S_N_1 lipid tails than the L21 model is consistently limited to positive curvature values.

### 3.4 Monolayer Pivotal Plane

The pivotal plane of a curved lipid monolayer corresponds to a surface within its core, along which the area per lipid does not change with curvature. For a homogeneous and perfectly elastic sheet, such a plane extends exactly in its middle, demarcating volume elements that are subject to compression and expansion in response to bending.^74^ In the case of lipid leaflets, different compressibility of polar heads and hydrocarbon chains leads to a shift of the pivotal plane toward the lipid–water interface, such that it is typically assumed to lie at , i.e., at 0.67 of the monolayer height,^74,75^ as measured from the bilayer midplane to the level of phosphate groups.

**Figure 5:**
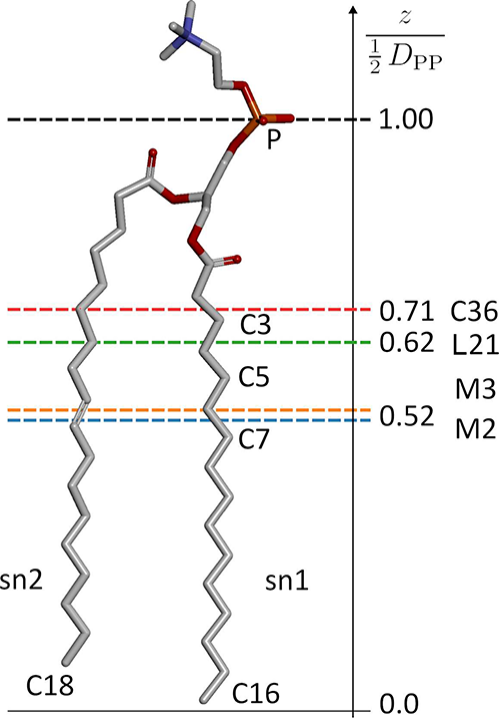
Schematic representation of POPC pivotal plane depth obtained for the considered force fields.

Our calculated pivotal plane location (Figure 5) indicates considerably better agreement with the above estimates in the case of both AA systems compared to CG systems. The obtained *z*_*p*_ values for the L21 and C36 force fields are 1.20 ± 0.01 and 1.37 ± 0.01 nm, respectively, which correspond to 0.62 and 0.71 of their respective POPC monolayer thicknesses. In the case of CG representations, *z*_*p*_ was found to be 0.986 ± 0.004 and 1.02 ± 0.01 nm for M2 and M3 force fields, respectively, corresponding to 0.51 and 0.53 of their thicknesses. These results indicate that, in contrast to AA representations, CG force fields produce a response characteristic to that of a simple elastic sheet. A similar observation has already been noted for the Martini DMPC model,^74^ leading to a conclusion that the CG hydrocarbon model has a limited capability to reproduce the adjustment of tail ordering in response to compression, and hence the pivotal plane shifts toward the middle of the monolayer to evenly distribute the bending penalty.

It will be of interest to investigate whether this issue could be remediated by the use of a softer nonbonded repulsion term than the one included in the standard, 12–6 type Lennard–Jones potential. Such a modification is introduced in the SPICA lipid force field,^24^ which utilizes a 9–6 type potential to describe the interactions between lipid beads. On one hand, this representation should indeed result in a more compressible membrane core, but on the other hand, as a similar softening is expected within the lipid head region, the actual location of the pivotal plane may not shift at all. In fact, it is plausible to assume that the difference in the compressibility of the lipid head and tail regions is inherently grounded in different physical mechanisms, with different proportions of entropy- and enthalpy-based contributions.^82^ In such a case, coarse grained models might just be too sparse to correctly capture the necessary nuances.

### 3.5 Monolayer Density and Interface Area Per Lipid

**Figure 6:**
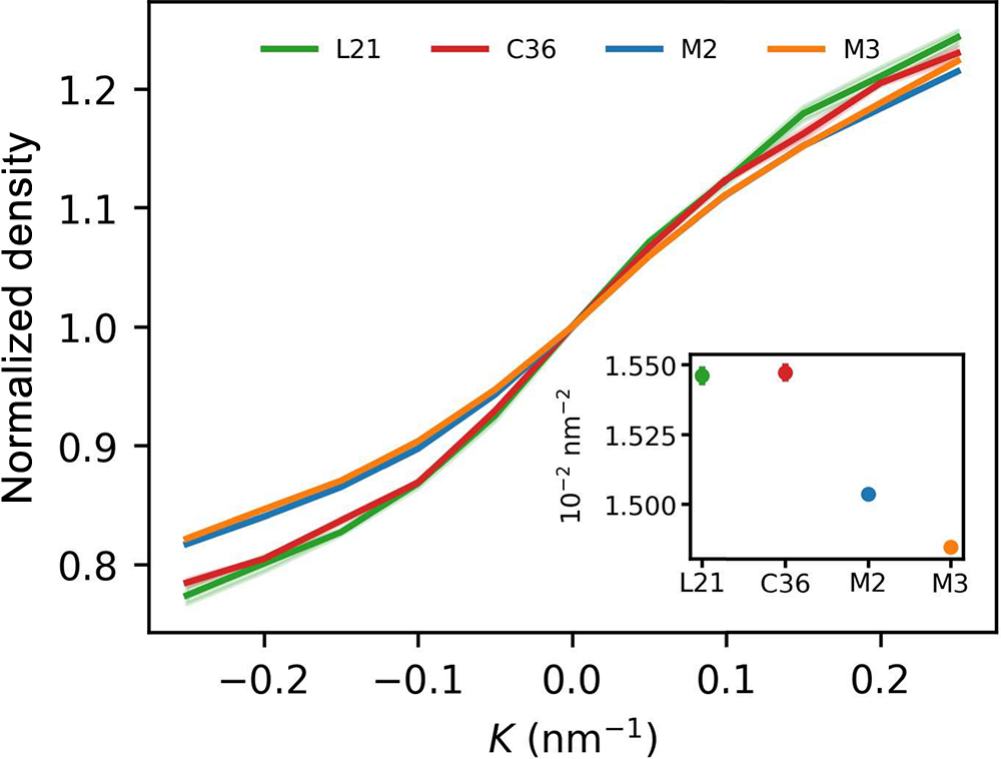
Monolayer area density as a function of curvature, normalized to 1 at *K* = 0 nm^–1^. Inset: absolute values at *K* = 0 nm^–1^.

The above characterized differences in the location of the pivotal plane for AA and CG representations translate to the way in which subsequent layers of lipid atoms respond to membrane bending. Linear density at the level of acyl chains termini is low in monolayer areas with a concave lipid–water interface and increases upon transition to regions with a convex lipid–water interface (Figure 6). In this respect, the L21 force field shows a slightly more pronounced response among the two AA models, possibly in line with its natively lower degree of acyl chains ordering compared to the C36 force field. In turn, both CG representations uniformly produce weaker responses than their atomistic counterparts, supporting the notion of limited capabilities to adjust to monolayer bending.

**Figure 7:**
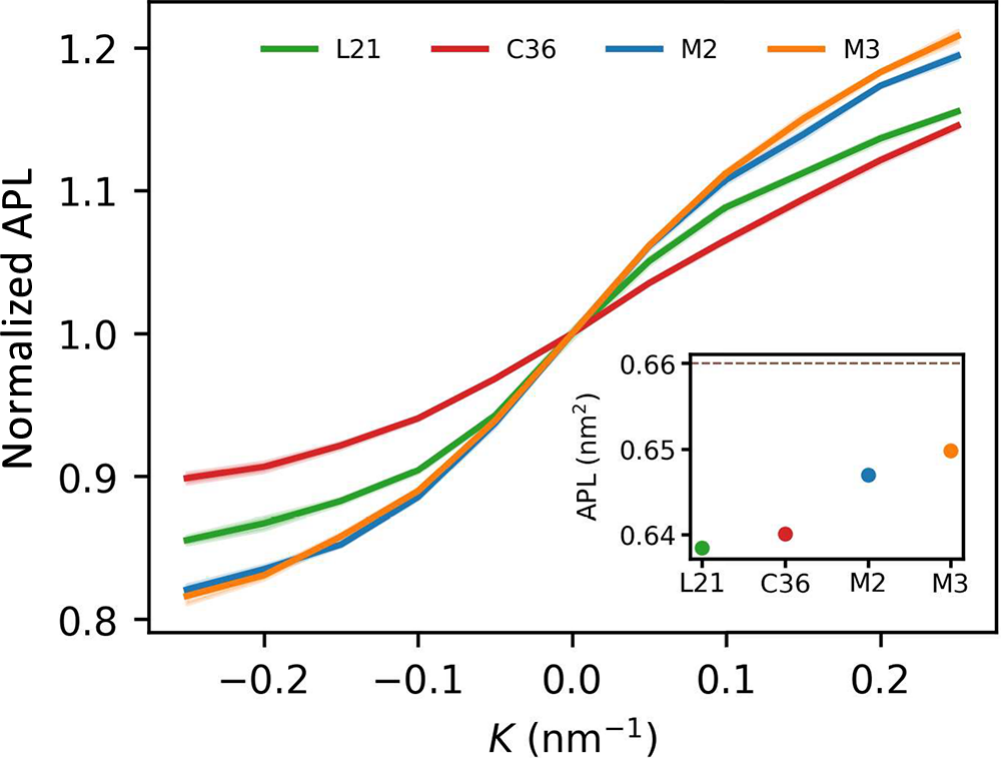
Changes in area per lipid in relation to curvature. Values are normalized to 1 at *K* = 0 nm^–1^. Inset: absolute values at *K* = 0 nm^–1^.

An opposite trend in the amplitude of curvature-related responses across the considered force fields can be seen in the case of area per lipid at the membrane–water interface (Figure 7). Here, both AA models produce a more moderate effect compared to the CG ones, in line with a shift of their pivotal plane closer to the monolayer surface. Intriguingly, unlike in the case of monolayer core density, there is a noticeable difference in lipid concentration at the interface between the L21 and C36 models, with the former showing stronger curvature dependence. Given the primary importance of the area per lipid among membrane parameters that are relevant for its biological functions, this discrepancy may have non-negligible influence on the way in which the force fields represent curvature-dependent orientation and sorting of membrane bound proteins during MD simulations.

### 3.6 Lipid Hydration

**Figure 8:**
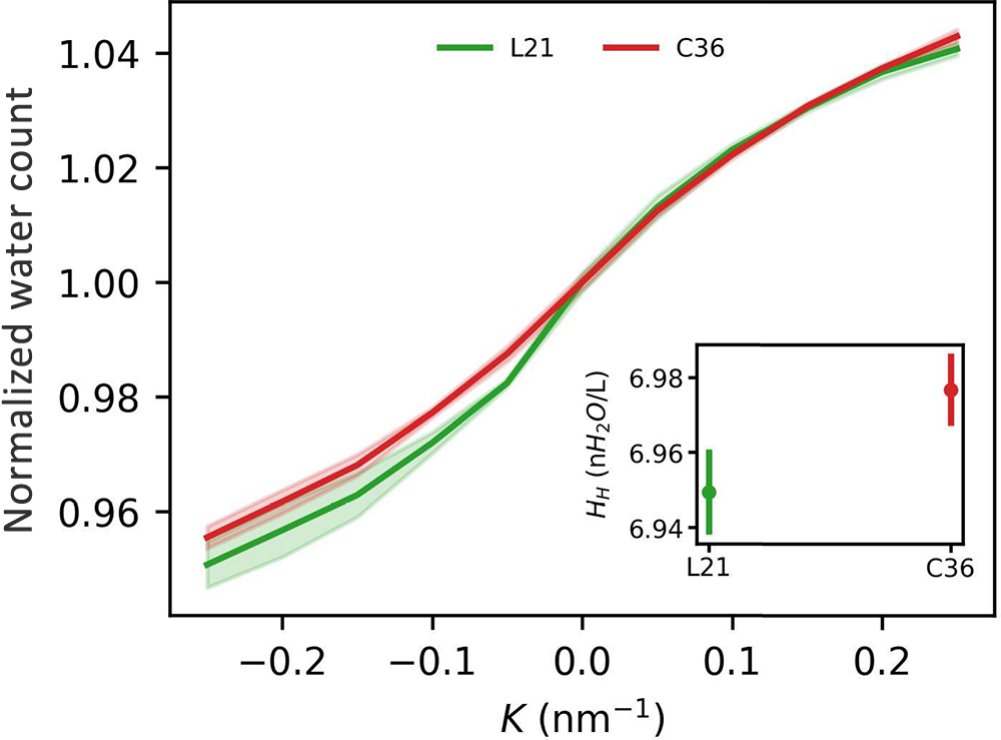
Number of water molecules within the first hydration shell of lipid heads as a function of curvature. Values are normalized to 1 at *K* = 0 nm^–1^. Inset: absolute values at *K* = 0 nm^–1^.

One direct consequence of bending-induced variations in lipid packing is curvature dependence of membrane hydration. A meaningful analysis can be conducted here only for AA models, since the CG representation is not capable of representing the physical molecular structure of the first hydration shell. Following our assumption of a 0.3 nm cutoff, POPC polar heads at a flat membrane–water interface were found to be hydrated on average by *H*_*H*_ ≈ 7 solvent molecules. As can be expected, the amount of water around lipid heads generally correlates with interface area per lipid (Figure 8), and the transition from *K* = −0.2 nm^–1^ to *K* = 0.2 nm^–1^ results in the recruitment of an additional 0.5 water molecule per lipid. Water depletion in a concave monolayer is marginally more pronounced in the case of the L21 force field, in line with its somewhat more compact interface as revealed by the APL assessment.

**Figure 9:**
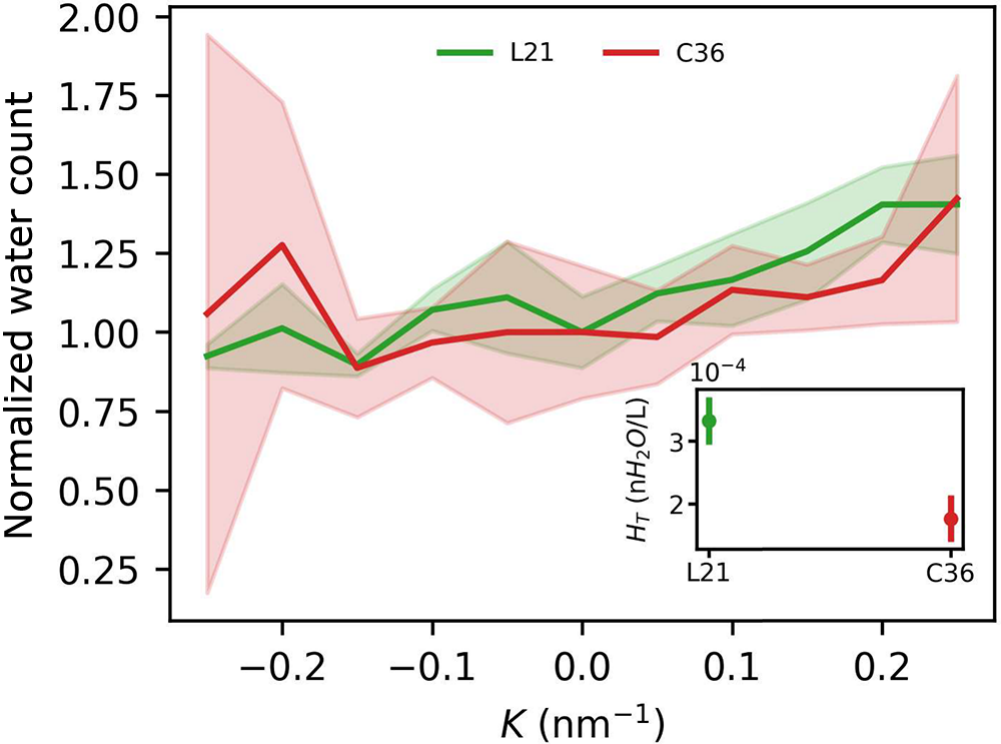
Number of water molecules within the first hydration shell of lipid tails termini as a function of curvature. Values are normalized to 1 at *K* = 0 nm^–1^. Inset: absolute values at *K* = 0 nm^–1^.

Intriguingly, hydration of lipid tails (Figure 9) tends to follow the curvature dependence observed for lipid heads, in spite of opposite density changes in response to bending within the membrane core and the interface regions mentioned above. Both AA force fields reveal greater availability of deeply buried water molecules in regions under a convex rather than a concave lipid–water interface. In this respect, the L21 model shows a slightly higher general hydration level, likely in line with the previously reported greater water permeability of Amber in comparison to the Charmm force field.^28^

### 3.7 Lipid Lateral Diffusion

In the case of AA systems, diffusion coefficients at *K* = 0 nm^–1^ are in fair agreement with values reported from experiments, as well as those obtained for flat membranes described by respective force fields.^28^ In turn, diffusion coefficients for both Martini lipids are ∼5 times larger than the ones for AA models, which can possibly be attributed to the smoother energy landscape inherent to CG representations.^83^

**Figure 10:**
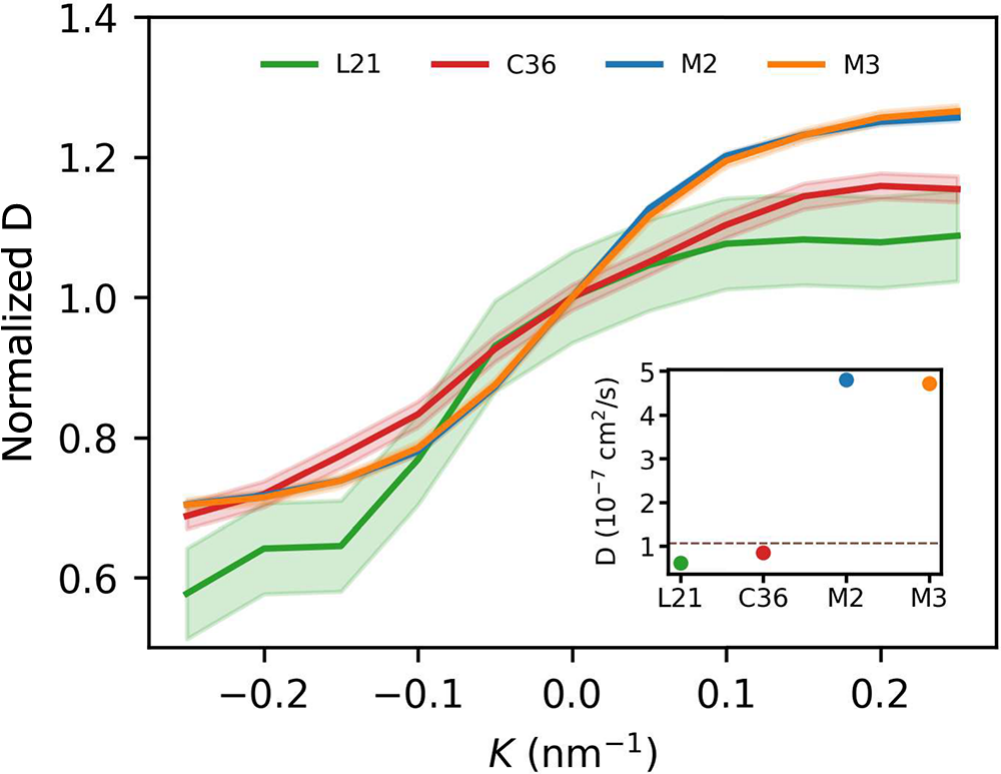
Lateral POPC self-diffusion coefficient as a function of curvature. Values are normalized to 1 at *K* = 0 nm^–1^. Inset: absolute values at *K* = 0 nm^–1^.

In all considered cases the rate of lateral lipid diffusion within the monolayer is found to significantly increase along with gradual transition from a concave to convex lipid–water interface (Figure 10). Of AA models, the L21 force field exhibits a more pronounced quenching of diffusion in concave areas, again in line with its more compact interface. Somewhat unexpectedly, the dynamics of C36 lipids appears to be also relatively enhanced in convex regions, in contrast to what might be expected from their greater ordering and smaller area per lipid in those areas compared to L21 lipids. The two Martini models yield virtually identical results to each other. Remarkably, the amplitude of relative changes of the diffusion coefficient is closer to that of AA models.

### 3.8 Mixed Membrane: POPC and Cholesterol

**Figure 11:**
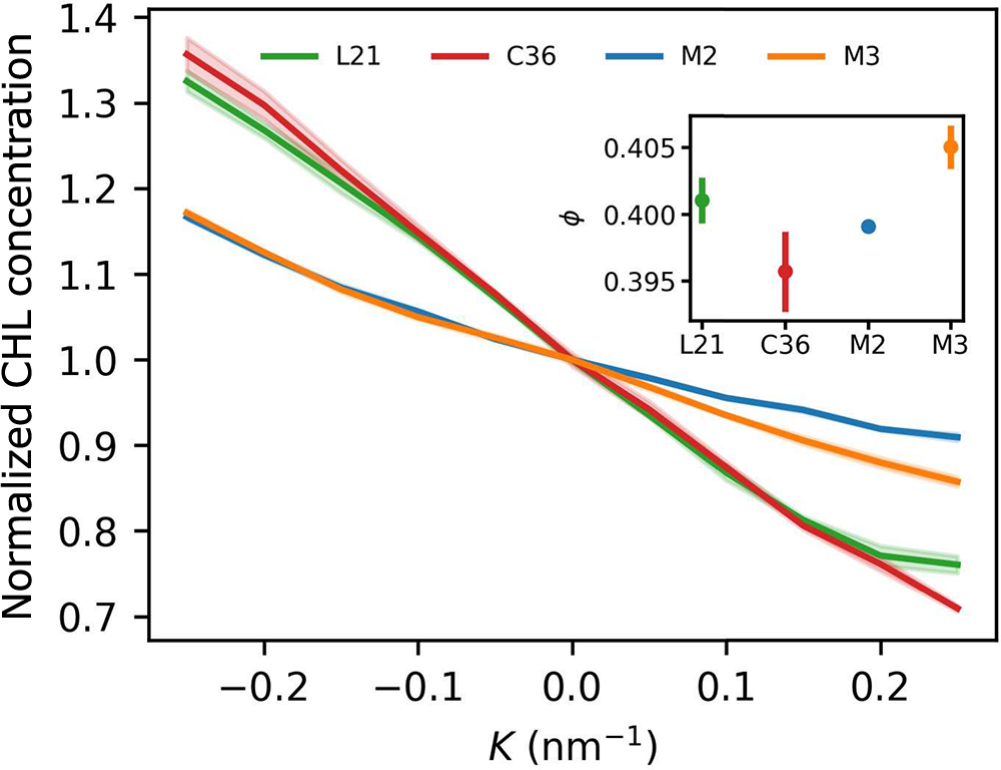
Changes in CHL mole fraction as a function of curvature. Values are normalized to 1 at *K* = 0 nm^–1^. Inset: absolute values at *K* = 0 nm^–1^.

Cholesterol is an essential component of lipid membranes, contributing to their structure, dynamics, and interactions with biological molecules. Owing to its chemical structure, with relatively extended hydrophobic and small hydrophilic parts, cholesterol is known to preferentially localize at monolayer regions with negative curvature.^73^ This tendency is clearly reproduced in our simulated membranes composed of POPC and CHL at 0.4 mole fraction (Figure 11). Notably, however, AA force fields are found to generate a significantly greater CHL concentration gradient in response to curvature compared to CG models. Among AA representations, the C36 force field shows a slightly more pronounced response compared to the L21 force field, whereas both Martini models produce identical results for concave monolayer regions and somewhat divergent ones at convex areas.

Assuming a simple, Helfrich-type curvature sorting model,^63,84,85^ one can derive a linear expression quantifying the relation between curvature and the logarithm of ratios of molar lipid concentrations at opposite monolayers (see the Methods, eq 5). The relative strength of curvature coupling for two lipid types *a* and *b* can then be obtained from the slope, *J*_*ab*_, of the fitted line.

**Figure 12:**
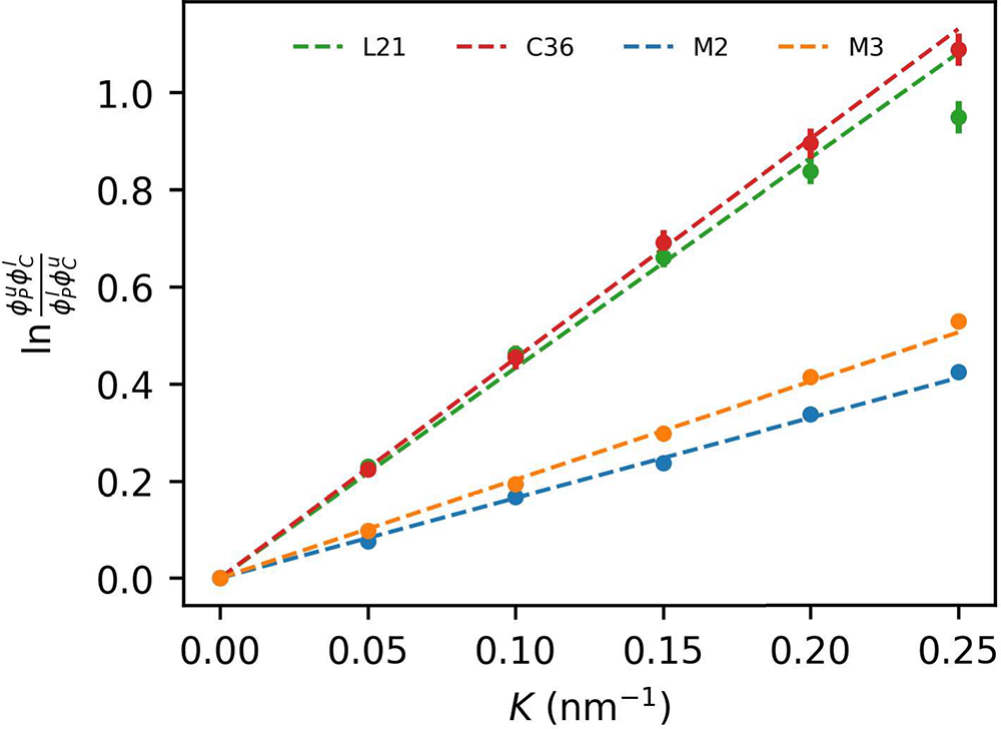
Enhancement ratio as a function of curvature (points) with a linear model fitted according to eq 6.

Our simulation data turns out to fit well into the above simple model, indeed producing nearly linear dependencies (Figure 12). As can be expected based on the analysis of concentration gradients, the relative strength of curvature–concentration coupling for CHL versus POPC lipids quantified by the *J*_*ab*_ parameter is much larger in the case of AA compared to CG models. If one assumes spontaneous curvature for POPC to be zero, then *J*_*ab*_ simplifies to .^84^ Further assumption of literature values for the membrane bending modulus, ,^86^ and area per CHL lipid in a CHL–POPC membrane, *S*_*b*_ = 0.30 nm^2^,^65^ allows assessing a semiquantitative estimate of CHL spontaneous curvature as . The numbers obtained for the L21 and C36 force fields, – 0.34 ± 0.01 and −0.38 ± 0.01 nm^–1^, respectively, are in good agreement with the reported CHL spontaneous curvature range of −0.34 to −0.49 nm^–1^.^77−79^ In turn, the values for the M2 and M3 models, – 0.137 ± 0.004 and −0.169 ± 0.005 nm^–1^, respectively, are too low, though some improvement in the newer force field version is apparent.

**Figure 13:**
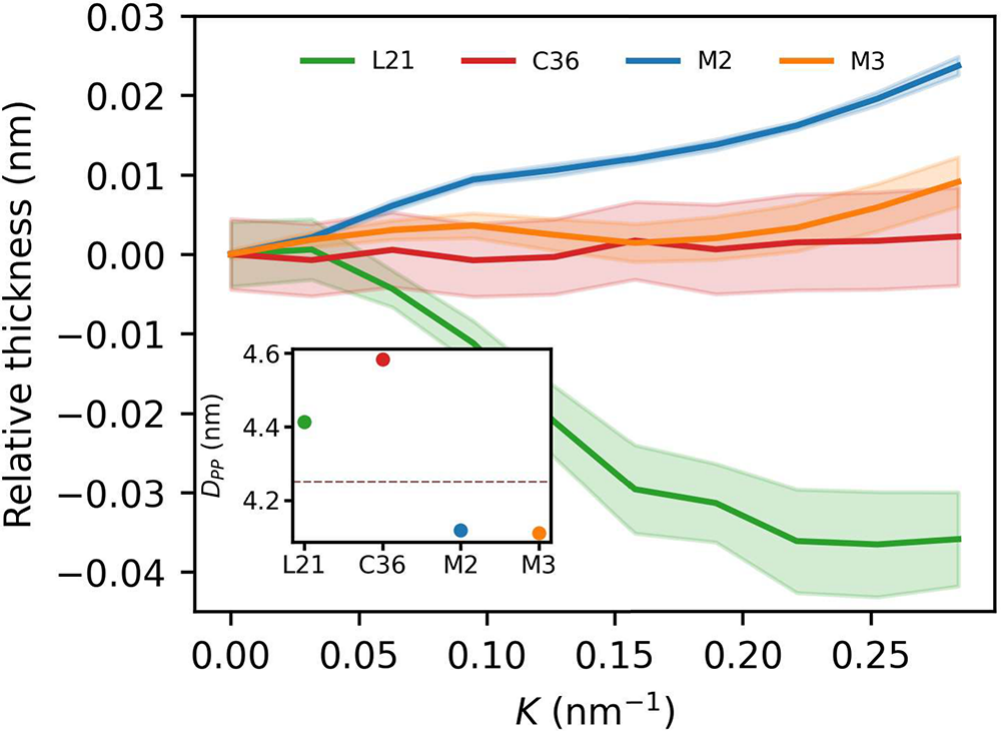
Changes in POPC–CHL membrane thickness in relation to curvature. All plots were shifted by reference values at *K* = 0 nm^–1^ (inset).

The presence of CHL, in particular among unsaturated phospholipids, is known to produce the so-called condensing effect,^87^ which manifests itself in a decrease of membrane area per lipid in relation to what would be expected from ideal mixing. This effect is accompanied by an increase in the overall membrane thickness and thus contributes to an important mechanism that governs CHL-dependent protein sorting within the membranes.^88,89^ In the case of curved lipid bilayers, thickening induced by an increased CHL concentration in the concave monolayer is expected to be compensated by thinning caused by relative CHL depletion in the opposite leaflet. In the case of the POPC–CHL mixture, such a compensation should be ideal, if one assumes that CHL concentration depends linearly on curvature and produces a linear response in membrane thickness.^36^ However, importantly, a quantitative assessment of this effect has not been undertaken to our best knowledge. Based on our simulations, the thickness of a two component POPC–CHL membrane indeed appears to be practically constant for the C36 and M3 force fields (Figure 13). Notably, however, L21 and M2 models show a qualitatively different behavior, with the AA force field producing an evident membrane thinning along with increasing curvature and the CG force field indicating membrane thickening. In the latter case, an increase in membrane thickness can be explained by deviation of ϕ_CHL_(*K*) from linearity (Figure S5) such that its concentration becomes relatively enhanced in curved bilayer regions. In the former case, however, no clear deviation from linear CHL concentration dependence on curvature, at least over *K* ∈ [−0.15, 0.15] nm^–1^, is observed (Figure S5), possibly pointing to peculiarities in membrane structural arrangement that warrant further investigation.

## 4 Conclusions

We conducted simulations of buckled POPC and POPC–CHL bilayers represented by four different force fields: two AA and two CG. Our comparison indicates that all models provide reasonable agreement with experimental values for major structural descriptors characterizing flat membranes. This consistency might be expected, given the kind of systems and observables that were used for their parametrization. Upon transition to relatively uncharted, curved regimes, quantitative differences, in particular between AA and CG representations, become evident. Unfortunately, an objective assessment of the models’ performance is hampered by rather limited experimental data concerning the changes of microscopic bilayer properties under curvature-generated stress. Nonetheless, the two independently developed AA models reveal a high degree of similarity in response to membrane bending, thus likely capturing reasonable baseline characteristics. This notion is further supported by the fact that both these force fields were found to produce satisfactory agreement with experimentally substantiated features such as the location of the monolayer pivotal plane or cholesterol spontaneous curvature radius. In turn, since the two CG models are closely related to each other, the mutually similar curvature dependence of most considered membrane parameters in their case is not surprising nor necessarily meaningful. It is of note, however, that the newer Martini model does not reveal an apparent tendency to produce membranes whose response to bending would better match that of AA models.

The primary source of discrepancies between AA and CG representations of curved membranes appears to originate from the limited capacity of the coarse grained hydrocarbon core model to capture a sufficiently rich response to lateral (de)compression exhibited during membrane bending. This affects the distribution of curvature-induced density changes between lipid head and tail regions, thereby causing a relocation of the monolayer pivotal plane from its anticipated depth, slightly below the glycerol backbone, to the midsection of the membrane leaflet. An important consequence of this shift, directly affecting the way in which membrane surface presents itself to surrounding biomolecules, is a much larger curvature dependence of interface area per lipid demonstrated by the CG models in comparison to the AA ones. This likely translates to a similar discrepancy in the magnitude of curvature-dependent lipid packing defects, which have been demonstrated to directly affect the sorting of surface-bound proteins.^90,91^ Accordingly, it may be expected that at the same level of membrane deformation, CG force fields may present a stronger sorting gradient for shallowly inserted protein motifs than the AA models. A parallel effect, but likely relevant for bilayer interaction with transmembrane domains, involves markedly restricted range of acyl chain ordering in response to membrane bending exhibited by lipids described by the Martini models. In this respect, it remains to be verified how and to what extent the organization of the membrane core affects protein sorting.

Given the rather small difference in bending-related modulation of bilayer thickness, the above discrepancies are likely the major factors determining the way in which AA versus CG models represent curvature sorting of membrane interacting compounds. To this end, our study reveals a substantial, 2-fold difference in curvature-induced cholesterol concentration gradients between the two considered classes of force fields. Whether it originates from the differences in POPC organization or intrinsic properties of the cholesterol model warrants further exploration; however, the introduction of a more complex cholesterol representation in the Martini 3 force field apparently brought only a modest improvement with respect to the previous version.

A biologically significant aspect of computational studies involving nonflat membrane topography is the proper reproduction of curvature sensing by membrane-bound proteins. Quantitatively correct modeling of this phenomenon presents considerable challenges, as it necessitates capturing the details of curvature-induced membrane remodeling, protein–lipid interactions, and appropriate hydration of both components. Importantly, however, the curvature-dependent distribution of proteins along the membrane is experimentally accessible, opening the door for direct evaluation of force field performance. We believe that our study represents an initial step in this direction.

## Supporting Information Available

Simulated systems, membrane shape approximation, calculations of the pivotal plane, order parameters, and CHL distribution (PDF)
