# Supplementary material for: Exploring the Properties of Curved Lipid Membranes: Comparative Analysis of Atomistic and Coarse-Grained Force Fields
Source: J Phys Chem B. 2024 Jul 11;128(29):7160–71. doi: 10.1021/acs.jpcb.4c02310 (PMC11284798; doi:10.1021/acs.jpcb.4c02310)
Supplement: Supplementary file 1 — jp4c02310_si_001.pdf [file jp4c02310_si_001.pdf]

Exploring the Properties of Curved Lipid Membranes: Comparative Analysis of Atomistic and Coarse-Grained Force Fields.
Supporting Information.

Maria Domańska and Piotr Setny*
Centre of New Technologies, University of Warsaw, Banacha 2c, 02-097 Warsaw, Poland
E-mail: *p.setny@cent.uw.edu.pl*

1 Simulated systems

Table S1: Details of considered curved systems and simulations. L21 – Amber Lipid 21 force field, C36 – Charmm36m force field, M2, M3 – Martini 2 and 3 force fields. Note that the M2 and M3 models use water beads equivalent to 4 explicit water molecules.

parameter	L21	C36	M2	M3
POPC				
n POPC	440	440	440	440
n H_2O	31561	31560	7967	7982
n H_2O/lipid	72	72	18	18
L_x (nm)	23.67	24.24	23.67	23.86
L_y (nm)	4.58	4.99	4.96	5.01
production (µs)	2.6	2.6	11.0	11.0
analysis (µs)	1.0	1.0	5.0	5.0
POPC:CHL				
n POPC	350	340	376	376
n CHL	234	222	252	252
n H_2O	35310	35308	18970	18970
n H_2O/lipid	60	63	30	30
L_x (nm)	23.66	23.80	23.50	23.63
L_y (nm)	4.69	4.87	4.94	4.98
production (µs)	1.5	1.5	11.0	11.0
analysis (µs)	1.0	1.0	5.0	5.0

Table S2: Details of considered flat systems and simulations.

parameter	L21	C36	M2	M3
POPC				
n POPC	200	200	200	200
n H_2O	9030	9053	2204	2183
n H_2O/lipid	45	45	11	11
L_x (nm)	8.13	8.08	8.04	8.23
L_y (nm)	8.13	8.08	8.04	8.23
production (µs)	1	1	1	1
analysis (ns)	500	500	500	500

2 Membrane shape approximation

Membrane shape was approximated by a linear combination of n sinus functions with wavelengths chosen to fit into the x-dimension of the simulation box, L_x:

$$Z(x) = A_0 + \sum_{n=1}^{n} A_n \sin\Big(2\pi n \frac{x - x_n}{L_x}\Big), \tag{1}$$

In order to establish the number of necessary components we took into account two opposing effects that arise with increasing n. First is the ability of the fitted function to represent membrane surface, which can be quantified by the root mean square deviations (RMSD) of distances of atomic positions in xz-plane from $Z(x)$. Second is the tendency to capture short wave membrane fluctuations that are registered as spurious, locally high curvatures. The higher number of components obviously allows better shape approximation evidenced by lower RMSD (Fig. S1A), however, at the same time leads to an increase in the amount of surface area whose curvature falls outside the range considered for analysis (Fig. S1BC).

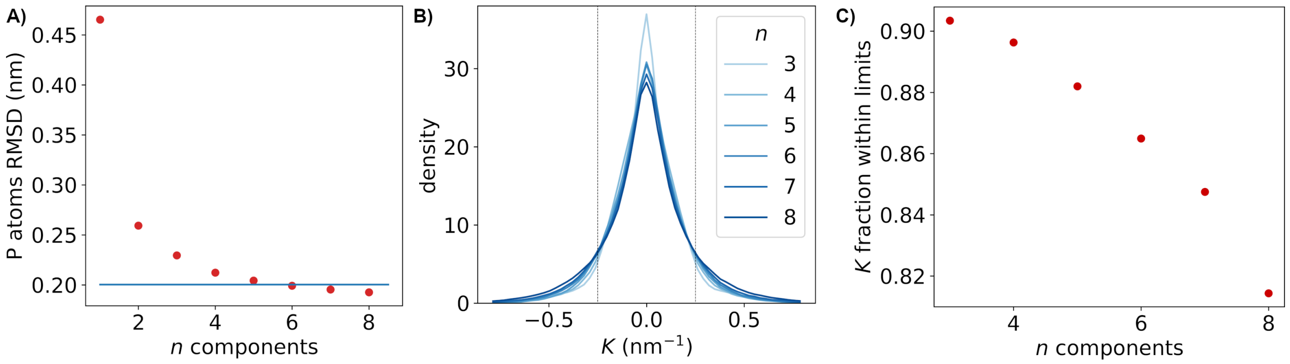

Figure S1: A) RMSD between phosphorus atoms positions and their fitted analytic curve as a function of the number n of considered sinus components. Blue horizontal line denotes an average RMSD for for phosphorus atoms positions within flat membrane, fitted to 3 nm $\times$ 3 nm square surface parallel to the membrane plane. B) The distribution of curvature probability density obtained with an increasing number of sinus components. Dashed lines denote $|K| = 0.25$ nm^{-1}, that is the limiting value of curvature used for analysis. C) The fraction of curvature probability density falling between $K = \pm 0.25$ nm^{-1}.

To arrive at the final number of $n = 6$ components, we assumed that sufficiently low RMSD would correspond to that obtained by approximating the surface of a flat 3 nm $\times$ 3 nm membrane patch by a planar surface, which turned out to be 0.2 nm (assessed based on flat membrane simulations). Under this assumption more than 85 % of membrane surface was mapped as having the curvature $K \in [-0.25, 0.25]$ nm^{-1}, which we deemed satisfactory.

In order to assess the effect of increasing the number of components used for membrane shape approximation, we compared the results obtained for bilayer thickness and surface area per lipid, that is two basic parameters characterizing transverse and lateral membrane parameters. Both sets of plots

(Fig. S2) reveal the same qualitative differences between atomistic and coarse grained force fields. We note that quantitative differences obtained for membrane thickness, are in the order on 0.03 nm (for the overall thickness of ~ 4 nm) that is similar to the one resulting from using different algorithms to assess the thickness of a flat membrane.

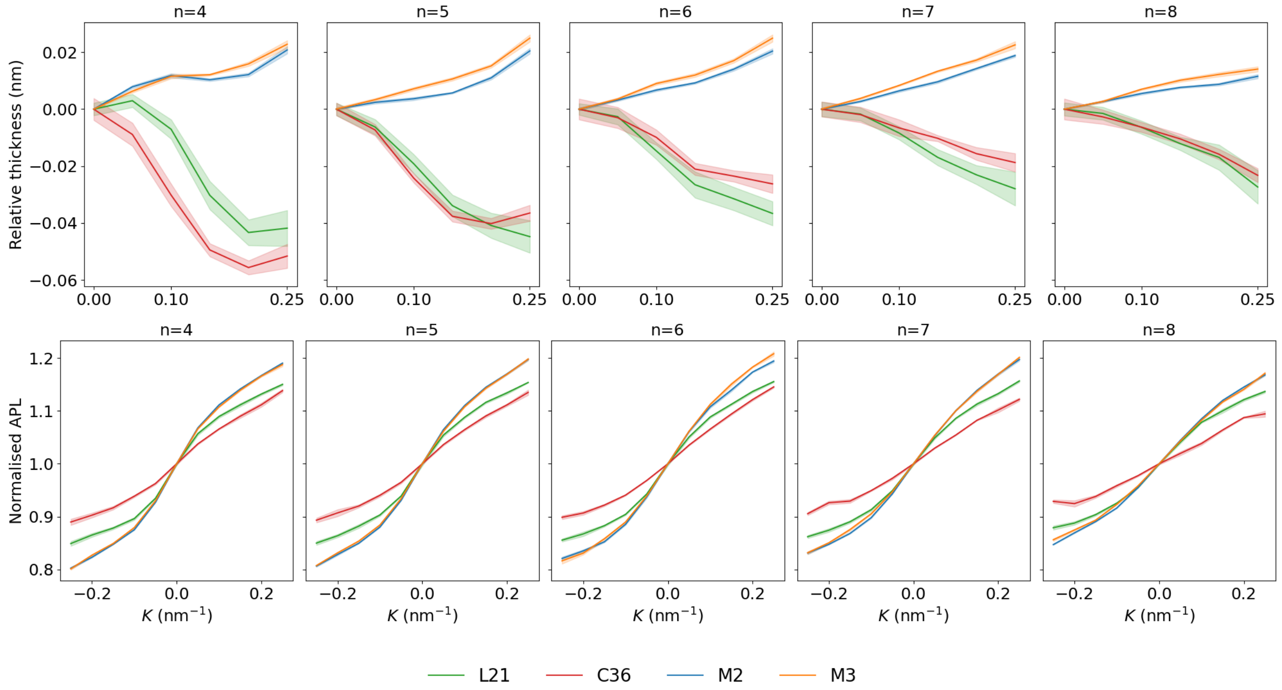

Figure S2: Relative membrane thickness (upper row) and normalized surface area per lipid (lower row) as a function of membrane curvature, obtained using membrane shape approximation based on n sinus components (Eq. 1).

3 Determination of monolayer pivotal plane

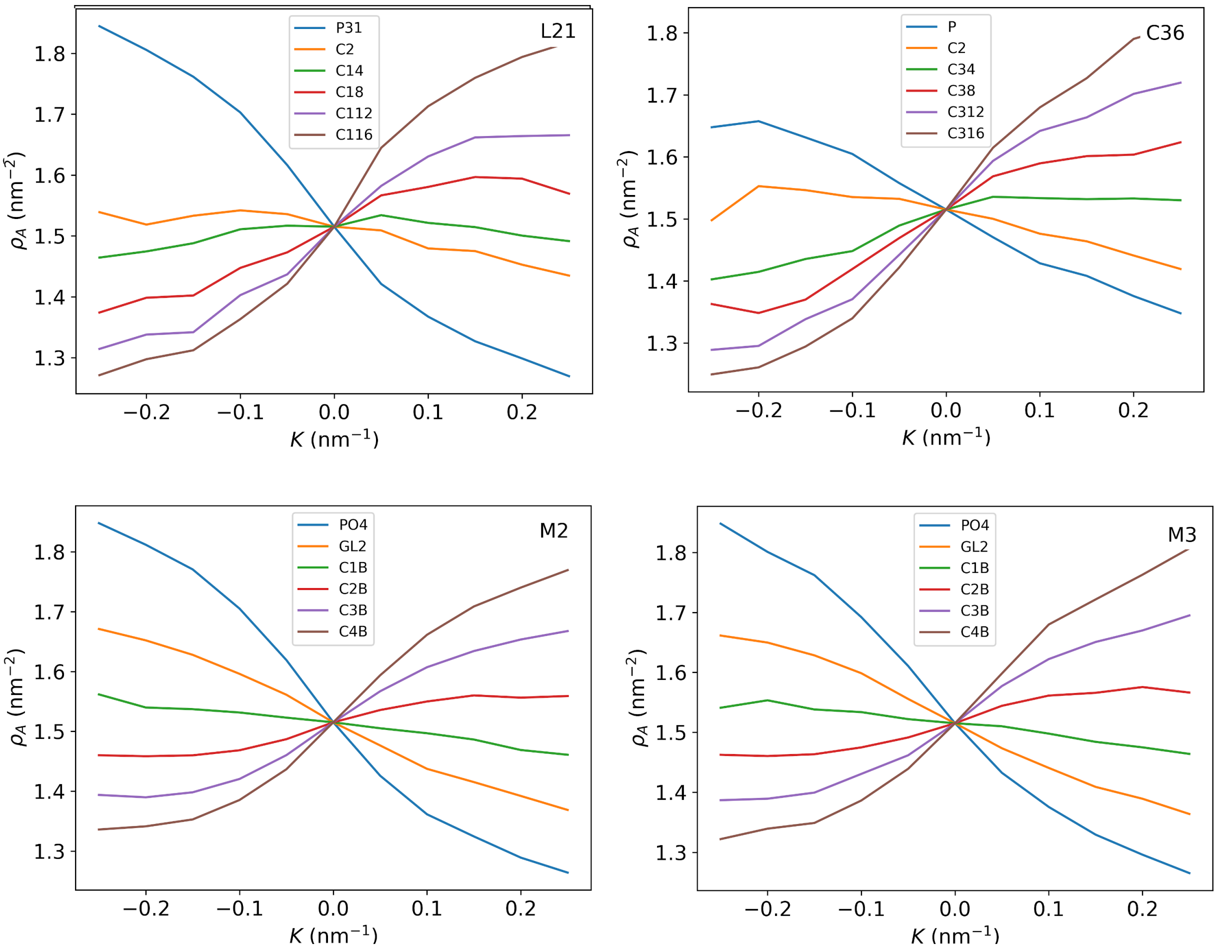

Figure S3: Sample monolayer atomic number densities along curved surfaces fitted to their positions in the xz-plane.

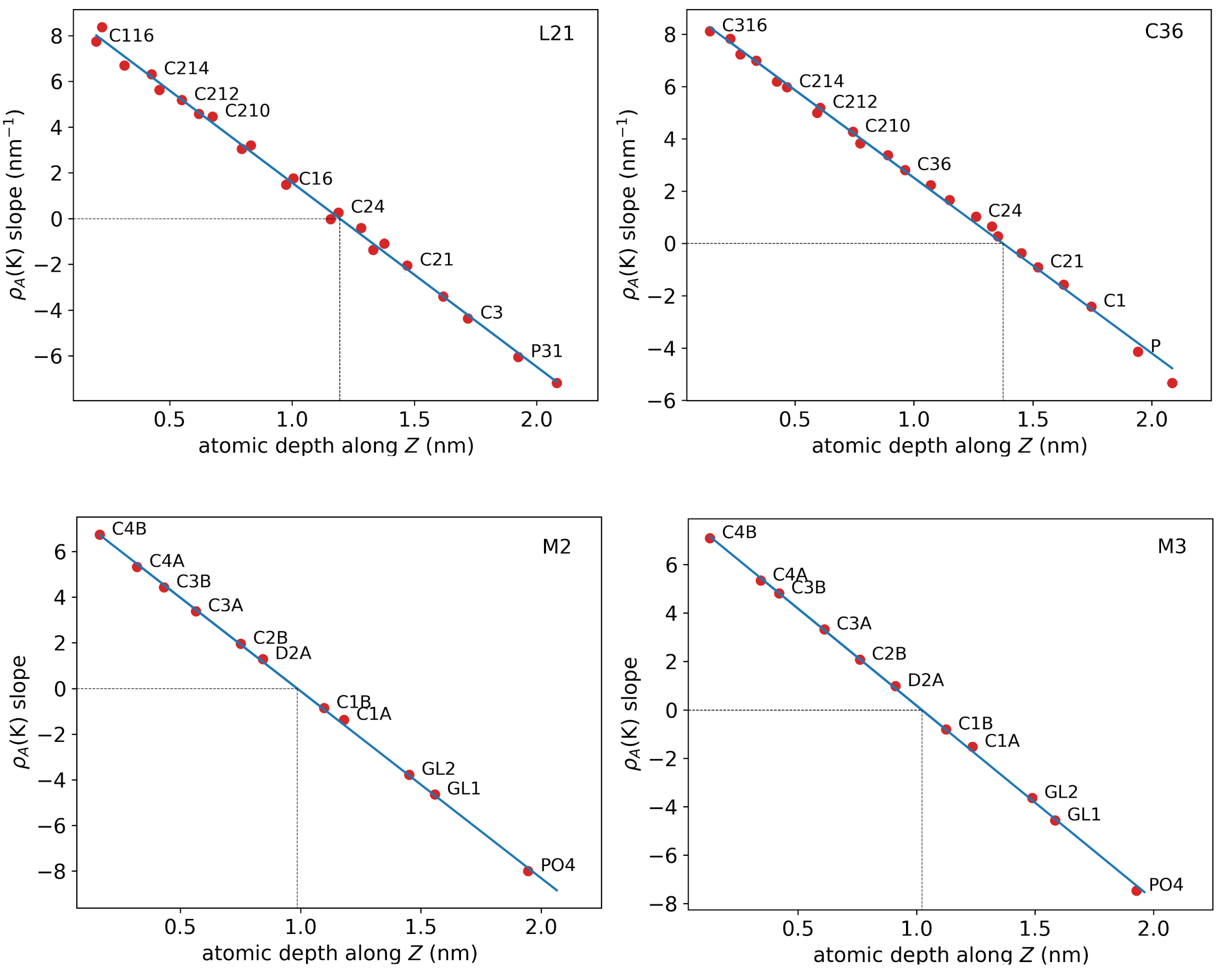

Figure S4: Slopes of atomic number densities obtained by linear function fit over $K \in [-0.15, 0.15]$ nm^{-1}, as a function of respective average atomic positions along membrane normal, z, with $z = 0$ corresponding to the bilayer midplane. Dashed line indicates the depth at which the slope would be 0, i.e. an approximate location of the pivotal plane, z_p.

4 Calculation of order parameters for atomistic force fields

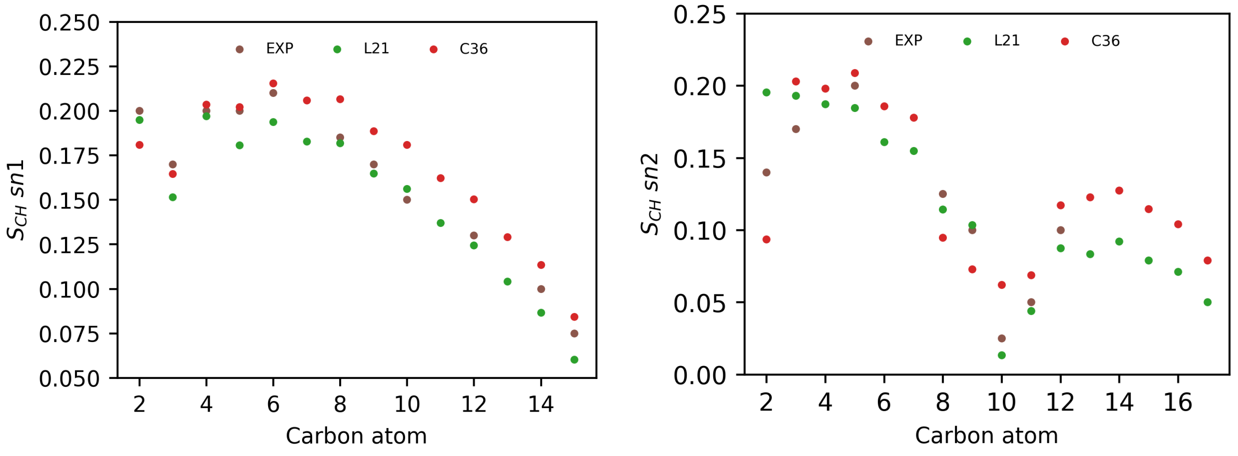

Figure S5: Left: mean S_{CH} values for flat systems - sn1 chain. Right: mean S_{CH} values for flat systems - sn2 chain. Experimental data are from Seelig et al. [1]

Table S3: Atoms used for calculation of lipid acyl chain ordering - S_{CC}

Force field	Atom selection
L21 SN1	C13 C17 C112 C116
L21 SN2	C23 C27 C212 C216
C36 SN1	C33 C37 C312 C316
C36 SN2	C23 C27 C212 C216
M2 SN1	C1A D2A C3A C4A
M3 SN1	C1A D2A C3A C4A
M2 SN2	C1B C2B C3B C4B
M3 SN2	C1B C2B C3B C4B

Table S4: Atoms used for calculation of hydration of lipid heads and acyl chains

Force field	Atom selection
L21 head	C3, C2, C1, O11, O21, O12, O22, C35, C33, C34, N31, C32, C31, O32, O34, O33, P31, O31
L21 tails	C215, C216, C217, C218, C113, C114, C115, C116
C36 head	O32, O31, C3, C2, C1, P, C11, C12, C13, C14, C15, O21, O22, O11, O14, O13, O12, N
C36 tails	C215, C216, C217, C218, C313, C314, C315, C316

5 Detailed parameters for buckled bilayers at $K = 0$ nm^{-1} and for flat membranes.

Table S5: Numerical values for membrane descriptors obtained for buckled membranes at $K = 0$ nm^{-1}.

parameter	L21	C36	M2	M3
S_{CH} sn1	0.15	0.17		
S_{CH} sn2	0.11	0.13		
S_{CC} sn1	0.379 ± 0.001	0.399 ± 0.003	0.279 ± 0.0002	0.276 ± 0.0003
S_{CC} sn2	0.345 ± 0.001	0.377 ± 0.001	0.344 ± 0.0003	0.371 ± 0.0003
D_{PP} (nm)	3.859 ± 0.002	3.899 ± 0.004	3.876 ± 0.001	3.834 ± 0.001
APL (nm^2)	0.6390 ± 0.0002	0.6400 ± 0.0004	0.6469 ± 0.0003	0.6970 ± 0.0003
z_p (nm)	1.20±0.01	1.37±0.01	0.986±0.004	1.02±0.01
H_{H}	6.945 ± 0.01	6.977 ± 0.01		
H_{T}	0.0003 ± 0.0004	0.0002 ± 0.0004		
D (10^{-7} cm^2/s)	0.62 ± 0.04	0.85 ± 0.02	4.81 ± 0.03	4.71 ± 0.03
ϕ_{CHL}	0.401 ± 0.002	0.396 ± 0.003	0.399 ± 0.001	0.405 ± 0.002
PC:CHL D_{PP} (nm)	4.413 ± 0.006	4.584 ± 0.006	4.117 ± 0.002	4.110 ± 0.001

Table S6: Numerical values for membrane descriptors obtained for flat POPC membranes.

parameter	L21	C36	M2	M3
D_{PP} (nm)	3.85 ± 0.015	3.95 ± 0.02	3.92 ± 0.02	3.88 ± 0.02
APL (nm^2)	0.65 ± 0.001	0.65 ± 0.01	0.66 ± 0.10	0.66 ± 0.01
D (10^{-7} cm^2/s)	0.52 ± 0.04	0.84 ± 0.04	6.12 ± 0.30	5.32 ± 0.28
S_{CH} sn1	0.15 ± 0.04	0.17 ± 0.04		
S_{CH} sn2	0.11 ± 0.06	0.13 ± 0.05		

6 Linearity of curvature-dependent cholesterol concentration

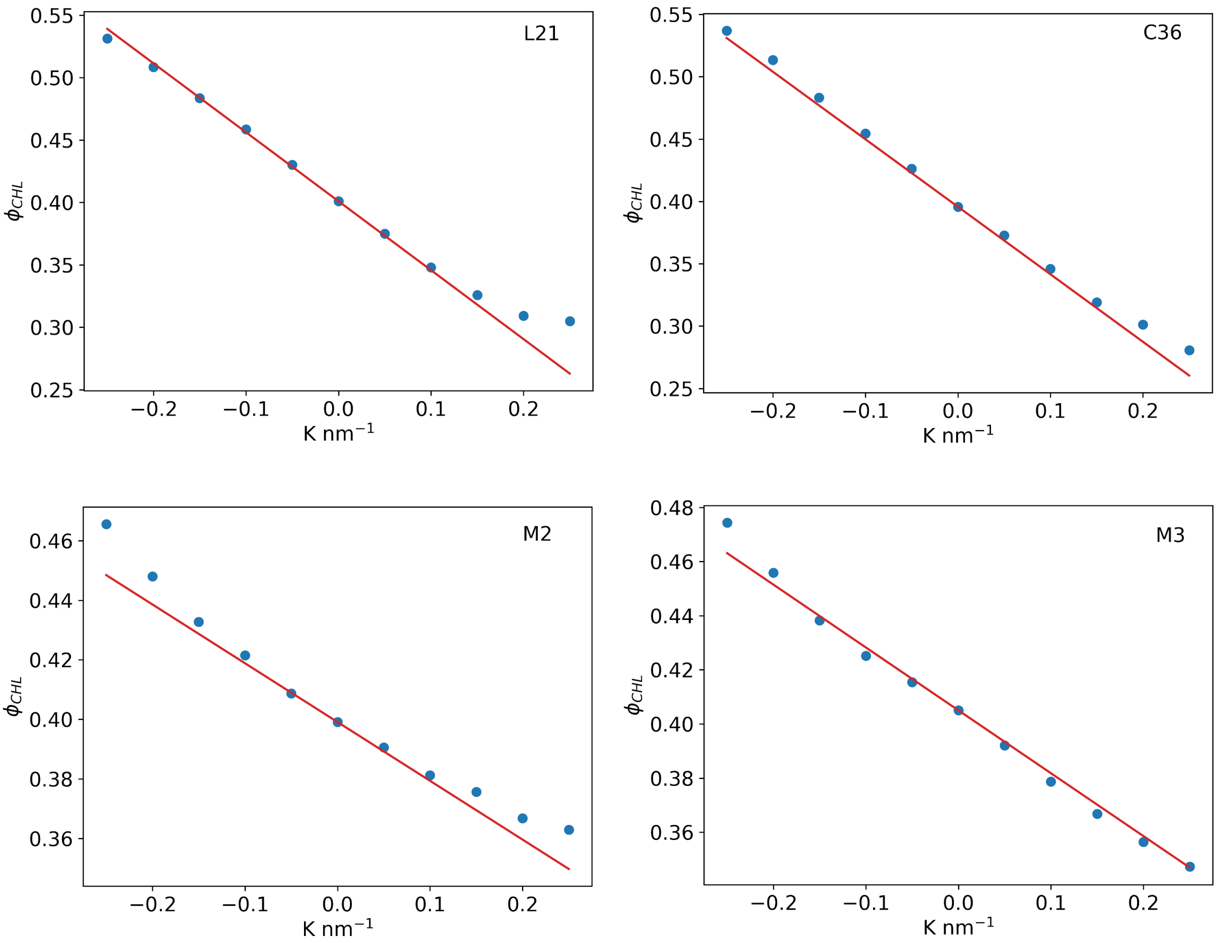

Figure S6: Cholesterol mole fraction, ϕ_{CHL}, as a function of curvature. For the sake of visual inspection, a linear model, $\phi(K) = aK + \phi(0)$, was fitted over $K \in [-0.1, 0.1]$.

References

[1] Joachim Seelig and Nada Waespe-Sarcevic. Molecular order in cis and trans unsaturated phospholipid bilayers. *Biochemistry*, 17(16):3310–3315, aug 1978.
